# The Fastest Saccadic Responses Escape Visual Masking

**DOI:** 10.1371/journal.pone.0087418

**Published:** 2014-02-05

**Authors:** Sébastien M. Crouzet, Morten Overgaard, Niko A. Busch

**Affiliations:** 1 Institute of Medical Psychology, Charité University Medicine, Berlin, Germany; 2 Center for Cognitive Neuroscience, Department of Communication and Psychology, Aalborg University, Aalborg, Denmark; 3 Cognitive Neuroscience Research Unit, Center of Functionally Integrative Neuroscience, MindLab, Aarhus University, Aarhus, Denmark; 4 Berlin School of Mind and Brain, Humboldt-University, Berlin, Germany; University of Groningen, Netherlands

## Abstract

Object-substitution masking (OSM) occurs when a briefly presented target in a search array is surrounded by small dots that remain visible after the target disappears. The reduction of target visibility occurring after OSM has been suggested to result from a specific interference with reentrant visual processing while the initial feedforward processing is thought to be left intact. We tested a prediction derived from this hypothesis: the fastest responses, being triggered before the beginning of reentrant processing, should escape the OSM interference. In a saccadic choice reaction time task, which gives access to very early stages of visual processing, target visibility was reduced either by OSM, conventional backward masking, or low stimulus contrast. A general reduction of performance was observed in all three conditions. However, the fastest saccades did not show any sign of interference under either OSM or backward masking, as they did under the low-contrast condition. This finding supports the hypothesis that masking interferes mostly with reentrant processing at later stages, while leaving early feedforward processing largely intact.

## Introduction

The interplay between feedforward and feedback processing is crucial for visual perception [1]–[4]. In psychophysics, this interplay has often been studied using visual masking [5], [6], and particularly object-substitution masking (OSM, also referred to as common-onset or four-dot masking). OSM occurs when a briefly presented target in a search array is surrounded by small dots that remain visible after the target disappears [2]. Interestingly, it is difficult for standard models of backward masking to account for the effect of such a trailing mask [2], [7] (but see [8]). DiLollo hypothesized that the “target plus mask” representation initially proceeds undisturbed through the feedforward sweep, and that OSM creates a mismatch between the reentrant signal representing “target plus mask” and the subsequent activity at the lower level representing the mask alone. Thus, in contrast to other forms of masking such as backward masking [9], OSM has been proposed to selectively disrupt reentrant processing while leaving the initial feedforward sweep intact [10].

Following these reports, the perceptual impairment caused by OSM has been used in numerous studies as a proxy for a selective disruption of reentrant processing: a task in which performance is impaired by OSM is assumed to require reentrant processing, while unimpaired performance in spite of OSM would indicate that only the first feedforward sweep is necessary to carry out the task [11]–[14]. For example, several studies demonstrated that even when the target cannot be consciously identified under OSM, its low-level, unbound stimulus features can be detected [12], [15] and trigger shifts of spatial attention [16], consistent with the notion that these processes do not require reentrant processing. Different task requirements would thus involve different types of processing: the first feedforward sweep might be sufficient to perform simple tasks such as simple feature detection, while more complex tasks seem to require additional reentrant processing. This conclusion is consistent with the notion that effects of visual masking depend on the criterion content, i.e. the particular task-dependent stimulus information that observers use to make judgments about the target [5].

However, the fact that OSM affects some, but not all tasks could be equally consistent with a weak, time-independent perceptual impairment that equally interferes with both feedforward and reentrant processing. The residual information surviving this weak impairment might indeed be sufficient to perform certain simple tasks (e.g. detection of simple visual features), but remains insufficient for the more complex ones (e.g. detection of feature conjunctions or semantically defined stimulus categories). Indeed, the notion that OSM selectively disrupts reentrant processing and that intact performance under OSM is due to intact feedforward processing is strongly debated [7], [8], [17], [18] and requires additional empirical support. Testing this claim requires observing a pattern of task performance that cannot be explained by a weak perceptual impairment occurring independently of when the behavioral response is initiated. Our hypothesis was that if OSM interferes selectively with reentrant processing while leaving the first feedforward sweep intact, then particularly fast, feedforward-driven responses should be unaffected by OSM.

**Figure 1:**
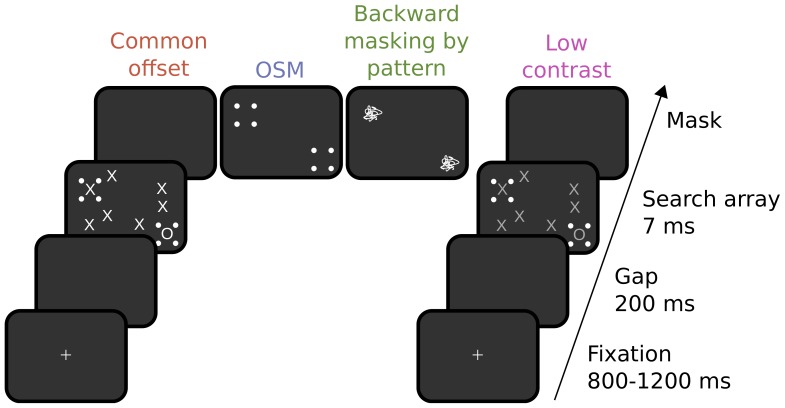
Schematic overview of the experimental paradigm. Observers made speeded saccades towards the location of the target (letter “o” surrounded by four dots). For the purpose of illustration, only 8 of 16 items of the search display are shown.

To test this prediction, we combined an OSM paradigm with a speeded saccadic choice task [19], [20] (Fig. 1). Observers were presented with a search array of letters (one O among multiple X) and were required to make a speeded eye movement to the side of the screen containing the target. We chose a saccadic choice task, rather than a manual task, because selective saccades towards target stimuli can be particularly fast. The minimal saccadic response time (i.e. the earliest time at which correct responses outnumber errors) can be as fast as 100–150 ms after stimulus onset [19], [20], while minimal manual response times would not occur before 250 ms [6]. Thus, the earliest selective saccadic responses are believed to be dependent on rapid visual processing, making this protocol an ideal tool for studying visual feedforward processing. In addition to a reference condition of high visibility in which the mask disappeared at the same time as the target (common-offset), we used three different methods to reduce target visibility: OSM, backward masking by pattern, and a global contrast reduction of the search array items. The low-contrast condition was used to test whether any kind of visibility reduction would yield the same effect as OSM and backward masking. Specifically, we matched the three low-visibility conditions for overall performance and tested whether OSM and backward masking, but not low contrast, leave particularly fast responses unaffected. Our results showed that observer’s performance was generally impaired by these three manipulations. However, the accuracy of the fastest saccadic responses in particular was totally unimpaired in the OSM and backward masking conditions. By contrast, in the low-contrast condition, both fast and slow saccades were affected. Accordingly, this indicates that the two types of masking left the initial feedforward processing relatively intact, while selectively disrupting later reentrant processing.

## Materials and Methods

### Participants

Four observers participated in the study (the first author and three naïve observers; aged 24 to 30 years; two males; two left-handed) after giving signed informed consent. All observers had normal or corrected-to normal vision. The experimental protocol was approved by the ethics committee of the German Psychological Association (DGPs).

### Apparatus

Observers sat in a silent and dimly lit room at a viewing distance of 57 cm. To improve eye tracking accuracy, a chin-rest was employed to maintain a stable head position and restrict head movements. We presented stimuli on a gamma-linearized 21 EIZO CRT monitor (screen resolution: 1024×768 pixels; refresh rate: 140 Hz) using MATLAB (MathWorks) with the Psychophysics Toolbox 3 [21]. Eye movements were recorded using an EyeLink 1000 Desktop Mount (SR Research). A 13-point calibration was performed before each block of trials. This large number of calibration points was used to improve eye-tracking accuracy.

### Procedure

The procedure combined a standard OSM paradigm with a 2AFC saccadic choice task [19], [20] (Fig. 1). A white fixation cross appeared in the center of the monitor for 800 to 1200 ms, followed by a 200 ms gap. A search array was then flashed for one frame (7 ms), consisting of the display of 16 letters (one “O” target and 15 “X” distractors, each covering 0.83°×0.83° of visual angle), presented at random locations in two 7×4 virtual arrays (covering 9.96°×11.62° in each hemifield). The target and one lure (distractor randomly selected in the opposite hemifield) were surrounded by a set of four white dots (0.21° each), centered on the imaginary corners of a 0.83°×0.83° square surrounding the letter. The following frames varied by condition (four equiprobable conditions, randomly interleaved in each block): (i) a reference condition with common offset of mask and search array; (ii) object-substitution masking (OSM), where the four dots remained on the screen for 300 ms after the offset of the search array; (iii) backward masking where a random pattern of straight and curved lines replaced the target and lure stimuli and remained on screen for 300 ms after the offset of the search array; (iv) a low-contrast condition, which was identical to the common-offset condition, but where the contrast of all letters was adjusted, so that observers performance was comparable to that in the masking conditions. Participants had to make a saccade, as quickly and as accurately as possible, to the side containing the target item. Each trial was followed by a 1000 ms blank intertrial interval. Each observer performed 40 blocks of 96 trials, except one of the naïve participants (observer 3) who performed 55 blocks.

### Performance Adjustment

A pilot study involving observers 1 and 2 tested the magnitude of the OSM-induced performance impairment. In the pilot study, stimulus contrast in the common-offset condition was adjusted by a staircase procedure (QUEST [22]) to yield 82% accuracy. Using the same contrast in the OSM condition yielded an accuracy of 58.4% (observer 1) and 63.4% (observer 2).

In the main experiment, we thus used a separate staircase for each condition throughout the entire experiment to adjust the contrast of the search array to fix each observer’s accuracy to 82% for common-offset and 60% for the three low-visibility conditions. This procedure reproduced the accuracy-reducing effect of OSM relative to common-offset (as observed in the pilot experiment) and ensured that accuracy was comparable in the OSM, backward masking, and low-contrast conditions. Note that the contrast values on which the adaptive procedure converged during the main experiment differed for each observer and condition (Fig. S1).

### Saccade Detection and Data Preprocessing

In the main experiment, saccade detection was performed off-line using Eyelinks built-in algorithm with standard thresholds for velocity (30°/s), acceleration (8000°/*s* ^2^) and motion (0.15°). For each trial, the onset of the first saccade after stimulus onset was considered as the saccadic reaction time (SRT). All trials with SRT faster than 70 ms were considered fast outliers (anticipations) and were discarded. Slow outliers were detected using an adjusted boxplot rule along with a robust skewness estimator [23]. We analyzed only the trials that occurred after the QUEST procedure had arrived at a stable estimate of the stimulus contrast necessary for 82% and 60% accuracy, respectively. Based on these criteria, data analysis included a total of 36,040 trials (91.9% of all collected trials). The preprocessed dataset used for analysis can be found in Dataset S1. We published the code used for data analysis and visualization under: https://github.com/scrouzet/MiniMask_Eye.git.

### Data Analysis

**Figure 2:**
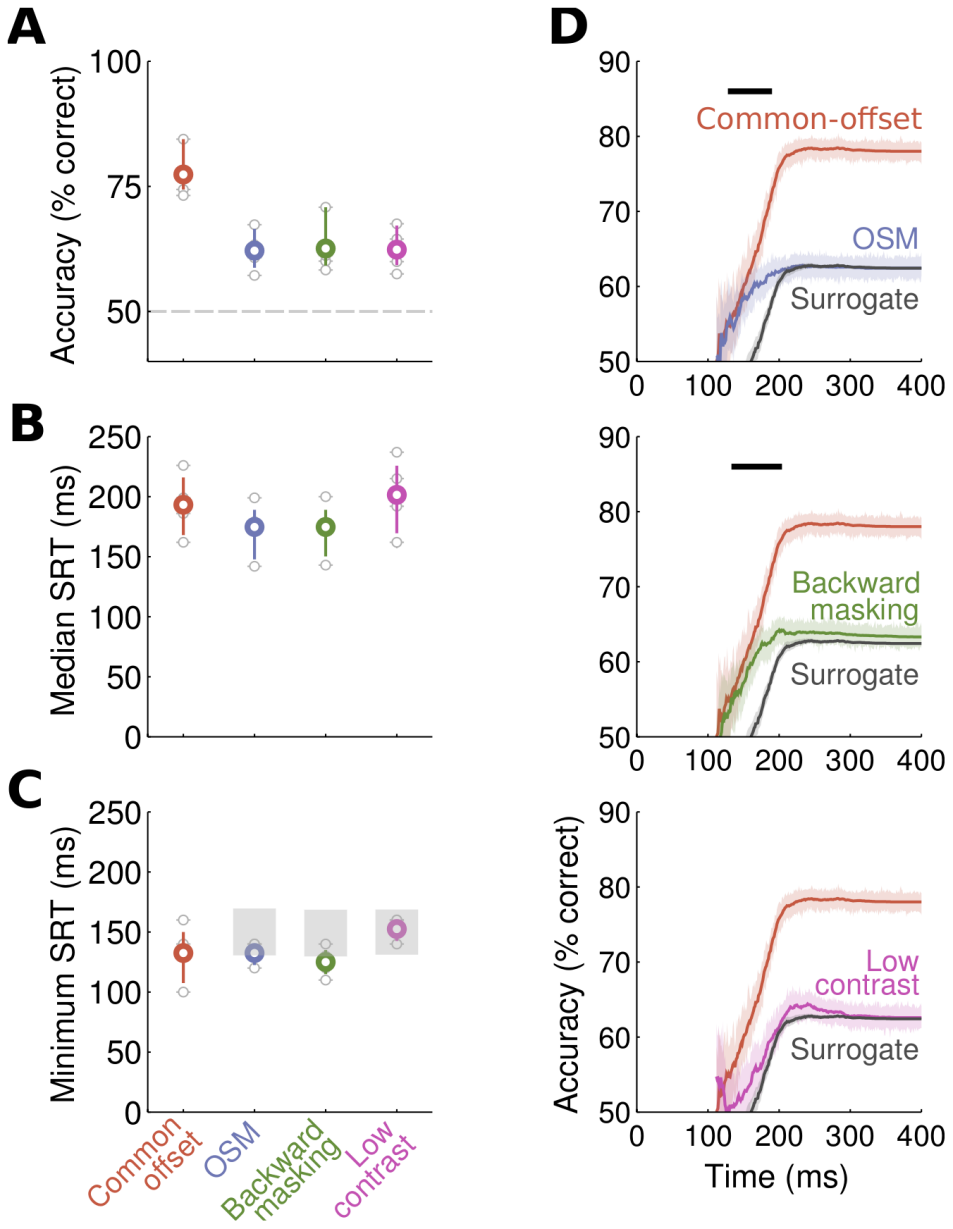
Accuracy and saccadic reaction times (SRT) results. (**A**) Accuracy averaged across observers (colored circles) in the four conditions and corresponding 95% CI. Light gray circles indicate results of individual observers. Note that target contrast was adjusted in the three low-visibility conditions to yield 60% accuracy. (**B**) Average and single-observer Median SRT. Same conventions as in (A). Median SRT were comparable in all conditions. (**C**) Average and single-observer Minimum SRT, computed as the fastest SRT at which accuracy was above chance level. Same conventions as in (A). For each low-visibility condition, superimposed light gray areas correspond to the 95% CI of the surrogate condition computed by swapping the correctness of trials from the common-offset condition until it matched the accuracy in the three low-visibility conditions. Minimum SRT for OSM and backward masking, but not for low-contrast, were significantly faster than expected based on the surrogate condition. (**D**) Accuracy time-course, obtained from the cumulative SRT distributions for correct and incorrect responses pooled across all observers (shaded areas correspond to 95% CI). For better readability, latencies before 110 ms are not displayed because not enough data were available to get a reliable measurement of accuracy. Horizontal black lines on top represent the time points at which the observed accuracy time-course in the three low-visibility conditions was significantly different from the surrogate time-course (obtained from the same resampling as in (C); non-parametric bootstrap test). Note that the fastest saccades under OSM and backward masking, but not for low-contrast, were as accurate as similarly fast saccades without masking (red trace) and significantly more accurate than predicted by the surrogate condition.

We computed cumulative SRT distributions to represent how accuracy depends on response speed. The time-points of this curve correspond to the time-ordered single-trial SRT. For each time-point of this curve, the accumulated accuracy corresponds to the proportion of correct responses that occurred up to this time-point. For example, the accuracy value at 200 ms indicates the proportion of correct responses for all SRT ≤ 200 ms. Since this measurement is very noisy for the first time-points, where just a few SRT were observed, we only show the curves after 110 ms in Figure 2.

To assess the timing of the fastest saccadic choice responses, we computed minimum saccadic response times (minimum SRT) for each condition and observer. They correspond to the first time bin (of at least 5 consecutive 10 ms bins) in the SRT distribution in which correct responses significantly outnumbered errors [24] (χ^2^ test). Thus, minimum SRT are a measure of the earliest time at which stimulus information is available for selective behavior in a given task. 95% confidence intervals for every variable of interest were obtained through bootstrap (n = 500). Accuracy time-courses and corresponding 95% CI were obtained from the cumulative SRT distributions for correct and incorrect responses pooled across all observers.

## Results

### Accuracy

Overall accuracy was identical in the OSM, backward masking, and low-contrast conditions, but significantly lower compared to the common-offset condition (Fig. 2A, post-hoc tests corrected for multiple comparisons using a Tukey HSD criterion following a single-factor ANOVA with four conditions; F(3,12) = 9.43; p = 0.002). This result was expected because accuracy was controlled by a staircase procedure in order to compare response times between low-visibility conditions of comparable accuracy.

### Response Times

Median SRT were similar in all four conditions (Fig. 2B, F(3,12) = 1.02; p = 0.41). Minimum SRT (earliest saccade latency at which accuracy was above chance level) averaged across observers were in the range of previous reports using a similar protocol with natural scenes (Fig. 2C, from 125 ms in the backward masking to 152.5 ms in the low-contrast condition, see for example [19]). No main effect of the condition was found on minimum SRT (F(3,12) = 2.28; p = 0.13), indicating that although accuracy was drastically impaired under masking and low contrast, the earliest time at which information was available to perform the task was not changed significantly overall.

### Do Fastest Saccades Escape Masking?

The central objective of our study was to investigate whether masking effects are dependent on the time at which a behavioral response is initiated. Specifically: can the fastest saccades, initiated before the mask starts to interfere, escape this masking effect? To answer this question, we first compared the minimum SRT measurements from the three low-visibility conditions to those obtained from a surrogate condition that corresponded to the null hypothesis that the effect of masking is SRT-independent.

For each of the three low-visibility conditions, we created surrogate distributions that were derived from the common-offset data, but for which we matched the accuracy to that of the low-visibility condition. These surrogate conditions were computed by drawing 500 random samples of SRT from correct common-offset trials. On each random draw, some of these correct trials were arbitrarily labeled as “incorrect” such that the proportion of “correct” and “incorrect” trials in the surrogate conditions exactly matched the proportion of correct and incorrect trials in each low-visibility condition. For each random draw, the minimum SRT was calculated and 95% confidence intervals were obtained from the distribution of surrogate minimum SRT across the 500 random draws. By design, the surrogate conditions all showed the exact same performance impairment as the three low-visibility conditions. Importantly, due to the random draw, the impairment in the surrogate conditions was independent of response time and thus represents the null-hypothesis that masking impairs perception uniformly over time.

The first analysis compared the minimum SRT obtained experimentally in each low-visibility condition against the 95% confidence interval calculated from the surrogate distributions. We found that minimum SRT obtained under OSM and backward masking were significantly faster than minimum SRT in the surrogate conditions. By contrast, minimum SRT in the low-contrast condition were not different from surrogate minimum SRT (Fig. 2C). This finding allows to reject the null hypothesis and to conclude that masking effects are response time dependent and spare particularly fast saccades. In contrast, the effect of reducing stimulus contrast impaired performance uniformly over time.

### Accuracy Over Time

To look more precisely at variations of response accuracy based on the time at which the responses were produced, we designed a measurement that allowed us to estimate how accuracy evolved as a function of SRT. This analysis of the time-course of accuracy was performed independently for each condition (Fig. 2D), and was based on the cumulative distributions of SRT for correct and incorrect responses. In these curves, the accuracy value at a given time point (e.g. 200 ms) corresponds to the proportion of correct responses for all SRT shorter than this value (e.g. all SRT ≤200 ms).

As can be seen on Fig. 2D, fast responses (between 120–130 ms and 200 ms) under OSM and backward masking were as accurate as similarly fast responses without masking (i.e. as compared to the common-offset curve), and significantly more accurate than predicted by the surrogate data. In other words, the fastest responses under masking showed no sign of a masking-induced perceptual impairment, while the effect of masking was observed only at longer latencies. By contrast, the low-contrast condition conformed well with the surrogate condition across all time-bins indicating that a simple reduction of stimulus contrast did not selectively impair visual processing at particular times. Importantly for this analysis, all conditions showed a similar overall distribution of response times and differed only in the ratio of correct and incorrect saccades (see Fig. S2). Thus, the failure to find a similar sparing of fast responses in the low-contrast condition was not due to a smaller number of saccades in this time window.

## Discussion

According to our current understanding of the visual system and its functions, visual perception is based on the interplay between feedforward and reentrant processing. Visual information is initially passed from lower-level to higher-level visual areas in a feedforward sweep that enables a rapid extraction of visual features. Feedback from higher back to lower areas, reentrant processing, is deemed essential for more complex visual functions such as figure-ground segregation or feature binding [1], [12], [25], [26]. Moreover, visual awareness is thought to be dependent on reentrant processing and ongoing communication between higher- and lower-level visual areas [4]. According to DiLollo [2], the initial feedforward sweep can be thought of as a first perceptual hypothesis, while reentrant processing allows for matching this hypothesis with sensory data. Thus, in the case of OSM, the incoming visual information after target offset (“mask alone”) does not match the reentrant signal that is still based on the initial “target plus mask`” display, resulting in the conscious perception of the mask alone, rather than of the mask plus the target. Thus, OSM has been proposed to selectively disrupt reentrant processing while leaving the initial feedforward sweep intact [10]. While this conclusion has not gone uncontested [7], [8], [17], [18], it is still central to most interpretations of OSM [11]–[14]. In the present study, we aimed at providing direct evidence for the reentrant account of OSM by testing the time course of the OSM-induced perceptual impairment.

Does the accuracy of behavioral responses depend on the time when these response are initiated, such that particularly fast behavioral responses are unaffected by OSM? Such a finding would support the idea that masking does not interfere with responses initiated during the feedforward sweep. Note that this reasoning does not assume a feedforward stage that is devoid of any reentrant processing. In fact, it has been demonstrated that responses in monkey areas V1, V2, and V3 are modulated by feedback from area MT almost from the beginning of the response onset [26]. Rather, we assumed that the contribution of reentrant processing would be initially small and increase over time [1], [26].

Here, we found that OSM and backward masking impaired performance predominantly for slower saccades, while the fastest saccades under masking were as accurate as comparably fast saccades without a mask. Importantly, we demonstrate that this effect cannot be explained by a generic, time-independent performance impairment. These results provide strong support for the claim that OSM leaves early feedforward-driven processing intact while disrupting mostly late reentrant processing. By contrast, the performance impairment caused by a simple reduction of stimulus luminance was independent of response time, indicating that it affected unselectively all processing stages.

### How Similar are OSM and Backward Masking?

Interestingly, the fastest saccades could escape both OSM and backward masking. This finding is in conflict with the idea that backward masking, unlike OSM, unselectively impairs both feedforward and reentrant processing [10]. Rather, the finding concurs with other studies reporting that backward masking does not impair the accuracy of the fastest behavioral responses in a rapid natural scene categorization task [6], and that it affects only the late part of neural responses in human EEG [27] and monkey visual cortex [28], [29].

### Is the Disruption of Reentrant Processing Necessary to Account for OSM?

It has been recently proposed that disruption of reentrant processing might actually not be needed to explain the OSM effect. Francis & Hermens [8] argued that the results presented by DiLollo et al. [2] – the effect of mask duration and set size – are insufficient to demonstrate the role of reentrant processing, since these effects can be accounted for by traditional quantitative models of backward masking that rely on a purely feedforward mechanism. In a subsequent study [7], the same group put forward a more sensitive experimental procedure to test whether object substitution masking is due to an impairment of feedforward or reentrant processing. They tested a prediction derived from feedforward and reentrant models of masking about the shape of the masking function. This function is obtained by testing performance for different target-mask stimulus-onset-asynchronies (SOA). For a weak mask, all models of backward masking predict a U-shaped masking function such that performance is best at very short and very long SOA and masking is strongest at intermediate SOA. By contrast, the reentrant processing account predicts that strong masking should occur only for common onset of the target and the mask (SOA = 0). Francis & Cho demonstrated that masking functions obtained with four-dot-masking are not U-shaped, but conform with the predictions of the object-substitution model [7], ruling out a purely feedforward processing account of OSM.

Following a similar line of thought, Põder recently demonstrated that a feedforward model with attentional gating could account for the set-size-dependent visibility reduction due to OSM [17], but this model was not able to account for the type of masking functions obtained under OSM, either [30]. Another framework introduced recently, called object-updating, offers a more psychological-level explanation of the effect of common-onset masks on visibility [31], [32], and does not seem to stand in opposition to the reentrant account. It is based on the observation that a target can be protected from OSM if it can be represented as a distinct object from the mask, supporting the idea that OSM is at least partly interfering with processing at the object-level. Our finding that masking predominantly affects late behavioral responses is in line with the reentrant account of OSM [2], but is nonetheless also compatible with object-updating.

### Modulation of Effective Mask Duration by Saccadic Reaction Time

It is well known that the strength of OSM depends on the mask duration, with the largest performance impairment for mask durations ranging from 80–300 ms [33], [34]. As far as we know, the minimum duration required to achieve a significant effect has not yet been established. Nonetheless, OSM has been demonstrated for masks as brief as 45 ms [33]. It is important to note that the target duration in our experiment was much shorter than in most previous OSM experiments (7 ms here vs. usually 80 ms). Thus, it is likely that in our study OSM would occur with even shorter mask durations than in previous studies. This finding is crucial for the interpretation of our results. Indeed, one could argue that in a saccadic choice task, the *effective* mask duration (as opposed to the duration of the mask’s physical presence on the screen) might depend on the saccade latency. Trials with fast saccades might result in shorter *effective* mask duration than those with slower saccades. However, this issue is unlikely to constitute a serious confound for the effect we report here. As can be seen in Fig. 2, the fastest saccades for which target information was not impaired by the mask, occurred in the time range of 125–160 ms after stimulus onset. Of course, the time from stimulus onset up to saccade execution is not solely used for visual processing *per se*, and thus cannot be equated directly to the effective mask duration. Instead, this time interval comprises several processes, including (1) the time for the visual information to reach relevant sensory areas from which a discriminative signal can be read out and (2) once the decision has been made, the time to initiate the saccadic motor command. In such basic shape discrimination tasks, the areas likely to contain the relevant visual information are possibly V4 and posterior inferior temporal cortex [19], [20], where the neural response starts as early as 60 ms after stimulus onset [1]. The time required for saccade initiation has generally been considered in the 20–30 ms range [19], [20]. Thus, if information transfer and saccade initiation together account for approximately 90 ms of the time leading up to saccade onset, saccadic response times of 125–160 ms indicate stimulus processing, and thus effective mask durations, of 35–70 ms. According to previous reports manipulating mask duration [33], [34], masks of this duration can effectively produce OSM. We thus conclude that reduced efficient mask duration for rapid responses is unlikely to account for the effect we observed.

### Not all Trials can be Processed in a Single Feedforward Sweep

Why is the target not always identified during the feedforward sweep, allowing for fast responses on all trials? First, it is important to note that the brief stimulus presentation (7 ms) and the high speed of the responses leave almost no margin for error. Thus, one source of the trial-to-trial variability in response speed and accuracy is whether attention is immediately allocated to the location of the target rather than the location of the lure or a distractor since the masking leaves no time for reorienting. Another limit to target detection during the first feedforward sweep comes from the notion of internal processing noise. This internal noise, which is at the core of models of response time distributions and perceptual decision making [35], [36], varies across trials. This variability can account for the fact that physically identical stimuli can, but will not always, be processed through a single feedforward sweep. We hypothesize that only those trials in which the internal noise is very low will allow for rapid target detection during the initial feedforward sweep.

In sum, by studying how observers’ accuracy unfolds over time, we were able to present novel evidence supporting the view that OSM and backward masking disrupt mostly reentrant processing while leaving the initial feedforward sweep intact.

## Supporting Information

Figure S1 **Single-trial contrast levels tested for each observer in each condition.** A generalized linear regression (gamma distribution and reciprocal link function) was used to quantify, on a single-trial basis, the correlation between the contrast values of the search items and the observed SRT. Beta parameters were displayed in red when p<.05. All slopes were either non-significant or very close to 0, which suggests that trial-to-trial variations of stimulus contrast due to the staircase procedure did not account for the trial-to-trial variability of SRT. Note that the contrast values differed for each observer and condition. Importantly, the stimulus contrast used in the masking conditions never exceeded that in the common-offset condition. Thus, our finding that fast saccades are spared by masking was not confounded by a higher contrast in the masking-conditions that might have caused a speed-up of behavioral responses.
(EPS)

Figure S2 **Cumulative distribution of correct and incorrect SRT for each observer in each condition.** Importantly for the analysis of the time course of accuracy (see Fig. 2), the fastest saccades (whether correct or incorrect) occurred at comparable latencies in all conditions. In other words, the first bins to contain responses in the distributions of SRT were similar for all four conditions. This validates our finding by showing that the delayed minimum SRT in the low-contrast condition (Fig. 2C and D) was not due to a smaller overall number of fast saccades, but due to the smaller number of correct relative to incorrect saccades.
(EPS)

Dataset S1 (CSV)
